# Evidence of Influenza A in Wild Norway Rats (*Rattus norvegicus*) in Boston, Massachusetts

**DOI:** 10.3389/fevo.2019.00036

**Published:** 2019-03-14

**Authors:** Charles O. Cummings, Nichola J. Hill, Wendy B. Puryear, Benjamin Rogers, Jean Mukherjee, Jessica H. Leibler, Marieke H. Rosenbaum, Jonathan A. Runstadler

**Affiliations:** 1 Department of Infectious Disease and Global Health, Cummings School of Veterinary Medicine, Tufts University, Grafton, MA, United States; 2 Department of Environmental Health, Boston University School of Public Health, Boston, MA, United States

**Keywords:** wildlife disease, urban rodents, Orthomyxoviridae, novel host, influenza

## Abstract

Influenza A virus (IAV) is known to circulate among human and animal reservoirs, yet there are few studies that address the potential for urban rodents to carry and shed IAV. Rodents are often used as influenza models in the lab, but the few field studies that have looked for evidence of IAV in rodents have done so primarily in rural areas following outbreaks of IAV in poultry. This study sought to assess the prevalence of IAV recovered from wild Norway rats in a dense urban location (Boston). To do this, we sampled the oronasal cavity, paws, and lungs of Norway rats trapped by the City of Boston’s Inspectional Services from December 2016 to September 2018. All samples were screened by real-time, reverse transcriptase PCR targeting the conserved IAV matrix segment. A total of 163 rats were trapped, 18 of which (11.04%) were RT-PCR positive for IAV in either oronasal swabs (9), paw swabs (9), both (2), or lung homogenates (2). A generalized linear model indicated that month and geographic location were correlated with IAV-positive PCR status of rats. A seasonal trend in IAV-PCR status was observed with the highest prevalence occurring in the winter months (December-January) followed by a decline over the course of the year, reaching its lowest prevalence in September. Sex and weight of rats were not significantly associated with IAV-PCR status, suggesting that rodent demography is not a primary driver of infection. This pilot study provides evidence of the need to further investigate the role that wild rats may play as reservoirs or mechanical vectors for IAV circulation in urban environments across seasons.

## INTRODUCTION

Influenza A virus (IAV) is a single-stranded, negative-sense, RNA virus with a segmented genome that belongs to the family Orthomyxoviridae. It is a virus that has impacted human populations since the nineteenth century and likely earlier (Barry, 2004; Taubenberger et al., 2007). Pandemic viral outbreaks, the worst of which was the Spanish Flu of 1918, can kill millions of otherwise healthy people worldwide, while seasonal influenza kills thousands of people every year and causes billions of dollars in loss of productivity (Molinari et al., 2007). Influenza has a large host range, including domesticated and wild animals. In 2015, an outbreak of highly pathogenic avian influenza (HPAI) cost the U.S. poultry industry one billion dollars and resulted in the culling of 50 million turkeys and chickens (McKenna, 2015). While such HPAI outbreaks have required extreme control measures as a response, the threat of outbreaks has been continuous in the past 20 years. Aside from strains circulating in poultry (H5NX, H7N9, H9N2), infection with endemically-circulating strains of H3 is commonplace in horses and dogs (Parrish et al., 2015). Persistent IAV infection and circulation amongst swine primarily causes impaired growth and weight loss leading to economic losses for producers (Kothalawala et al., 2006).

Influenza infections have also been documented in numerous species of wildlife, with a growing interest in wild animals that overlap with human settlement and agriculture. Marine mammals, including seals, are known to be infected with both influenza A and B strains (Hinshaw et al., 1984; Puryear et al., 2016). Outbreaks occur periodically in seals, creating opportunities for exposure between seals, humans, and other wildlife that overlap along densely populated coastal margins (Runstadler et al., 2013). It is thought that wild birds, particularly migrating water birds belonging to the orders Anseriformes and Charadriiformes, are the reservoir host of IAV (Webster et al., 1992), and contact with waterfowl is a known risk factor for HPAI outbreaks in poultry (Shortridge et al., 2000). The increasing interface between wild and domestic birds, owing to the conversion of natural wetlands to agriculture, presents challenges for controlling the spread of IAV both in Eurasia and North America (Hill and Runstadler, 2016). Aside from seals and birds, however, relatively few studies have investigated the presence of IAV circulating amongst other wildlife, particularly species such as rodents that come into close contact with humans living in cities.

Wild, urban rodents are a ubiquitous but understudied species that may contribute to the epidemiology of influenza in urban environments. Climate change and milder winters across temperate regions contribute to growing urban rat populations (Atkin and Keizer, 2017). Rats thrive in the built environment allowing for frequent contact with humans and wild birds—both established as important hosts for influenza. Rats also have frequent contact with cats, which are known to transmit influenza to humans, albeit as an influenza host of minor importance (Belser et al., 2017). In addition, rodents harbor zoonotic pathogens like hantaviruses, *Leptospira* sp., arenaviruses, and others (Himsworth et al., 2014). In Vancouver^1^, antibiotic-resistant *E. coli* were found in roughly 5% of black and Norway rats, consistent with studies in German cities (Himsworth et al., 2015). In New York City, rats were found to carry *Leptospira* sp., *Bartonella* sp., Seoul virus, gastroenteritis-causing bacteria, as well as a number of previously uncharacterized viruses (Firth et al., 2014). Yet few studies have ever looked for influenza specifically amongst urban wild rodents. In Egypt and Hong Kong, evidence of IAV infection in wild rats and mice has been documented, albeit at a low frequency (Shortridge et al., 2000; Shriner et al., 2012; El-Sayed et al., 2013). In the US, those that have looked for IAV have typically been in the wake of outbreaks in rural settings such as poultry barns (Nettles et al., 1985; Shriner et al., 2012; Grear et al., 2016); however, rodents sampled in these studies were negative for IAV (Nettles et al., 1985; Grear et al., 2016).

The city of Boston, Massachusetts, located in the northeast of the United States is an ideal urban setting to study influenza in wild rodents, as Boston was recently identified as having the second highest level of rodent infestation among large cities as reported by the U.S. Census Bureau; American Housing Survey (2015). To identify the role of wild urban rodents in influenza ecology in the context of a city with significant human and rodent cohabitation, we evaluated the prevalence of IAV carriage among trapped wild rats in Boston over a 2-year period. To our knowledge, this is the first study to consider IAV among wild rodents in a major U.S. city.

## MATERIALS AND METHODS

**FIGURE 1 |:**
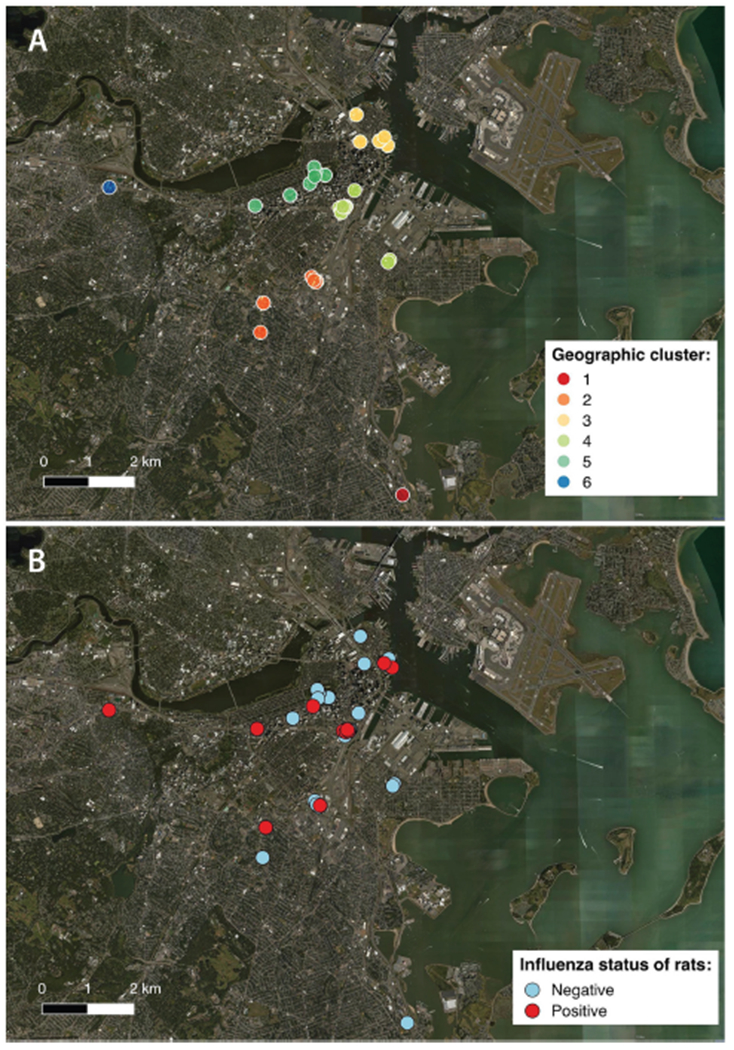
Locations of individual sampling sites within the City of Boston, color coded according to **(A)** the 6 geographical clusters (“geoclusters”) identified with k-means clustering, a process that grouped rat sampling sites according to geographic proximity based on latitude and longitude; **(B)** influenza A virus status of rats based on molecular screening (positive or negative).

This study was cross-sectional in design and aimed to recover rats from varying microhabitats (parks, alleys, etc.) across the City of Boston from December 2016 to September 2018 (Figures 1A,B). Individually bagged Norway rat carcasses were provided by the City of Boston’s Inspectional Services. Rodents were collected within 6 h of trapping and were transported on ice to the lab for immediate processing.

Necropsies were conducted aseptically under a laminar flow hood in a BSL-2 laboratory. Rats were sexed and weighed, and swabs of the oronasal cavities and paw pads were obtained using polyester swabs (Puritan, Maine, USA). Oronasal swabs were collected by swabbing the external nares followed by opening the mouth and inserting the swab at the back of the throat at the junction of the oropharynx and nasopharynx. Swabs were immediately placed in viral transport media (VTM: Remel, CA) and frozen at −80°C prior to screening for the viral RNA (Puryear et al., 2016). Lungs were then harvested aseptically, cryofrozen without media or in viral transport media, and stored at −80°C prior to processing.

Lung samples were homogenized for detection of viral RNA. An ~20 mg piece of lung tissue from each animal was placed into a prefilled 2 ml disruption tube with 2.8 mm stainless steel grinding balls (OPS Diagnostics, NJ) and 350 μl of AE Buffer (Qiagen, Germany) and 350 μl milliQ water. The tube then underwent bead-beating for 1.5 min in a cold room (4°C) prior to further processing.

Viral RNA was extracted from 50 μl of the swab samples in VTM and from lung homogenate samples using the Omega Mag-Bind Viral DNA/RNA kit (Omega Bio-Tek, Norcross, GA, USA) and a Kingfisher Magnetic Particle Processor (Thermo Scientific, Waltham, MA, USA). RNA was screened using qScript XLT One-Step RT-qPCR ToughMix (Quanta Biosciences, Gaithersburg, MD, USA) and analyzed for fluorescence on an ABI 7500 real-time PCR System (Applied Biosystems, Foster City, CA, USA) for a conserved IAV matrix gene segment (M) target, as previously described (Spackman et al., 2002). VTM was used for negative controls in both extraction and PCR steps. Influenza A/Puerto Rico/8/1934 was used as a positive control for the extraction step and extracted RNA from PR8 strain IAV served as a positive control for the PCR step. Samples producing cycle threshold (Ct) values ≤45 were considered positive for IAV RNA. This high Ct cut-off was deemed necessary given the trace amounts of virus associated with wild reservoir species and the potential for inhibitors from the raw sample to reduce detection of virus during PCR.

Positive samples were inoculated into the allantoic cavity of 10-day-old embryonated chicken eggs (ECEs) (Charles River, CT, USA), and incubated at 37°C for 72 h. RNA was extracted from 50 μl of amnio-allantoic fluid (AAF) and screened for the IAV M gene as described above. Whole genome sequencing was attempted on RNA from IAV positive AAF (Ct ≤ 45) and RNA from positive raw samples with a Ct ≤ 37 at the J. Craig Venter Institute in Rockville, MD, as previously described (Nelson et al., 2007). Repeated passage of the positive AAFs was not successful in boosting the viral concentration, reflected by RT-PCR Ct values on passage AAF (data not shown).

Data was analyzed using JMP (JMP®, Version 14.0 SAS Institute Inc., Cary, NC, 1989–2007). Descriptive statistics were used to summarize influenza PCR status (prevalence, 95% confidence intervals) stratified by demographic variables (sex and age).

To group sampling locations that were in geographic proximity, K-mean clustering was performed using latitude and longitude. Data were partitioned from 1 to 20 clusters using an iterative fitting process and the cubic clustering criterion was used to determine the optimal number of partitions or “geoclusters” for the study. K-means clustering indicated that 6 independent geoclusters (Figure 1A) was the optimal number of partitions for characterizing the distribution of sampling locations. Mapping of the sampling locations was performed using QGIS 2.18 (qgis.org) and color coded according to geocluster (Figure 1A) and influenza infection status (Figure 1B).

A generalized linear model (GLM) was used to assess associations between sex (categorical: male/female), weight (continuous: grams), sampling month (ordinal: Jan, Feb, May, June, Jul, Aug, Sep, Dec), and geocluster (categorical: 1–6) with influenza status expressed as a dichotomous result (i.e., positive/negative). Categories of variables with fewer than 5 data points were excluded from analysis. For instance, month (May, Dec), geocluster 1, and rats of unknown age or sex were all excluded prior to performing GLMs. An information-theoretic approach (Akaike’s Information Criterion [AIC]) was used to compare IAV status of rats under different *a priori* defined models relevant to the epidemiology of influenza transmission in wild animals.

To assess whether different specimen types (oronasal, paw, lung) had significantly different Ct values, data were analyzed using one-way ANOVA.

## RESULTS

A total of 163 Norway rats were trapped over the course of this study. Eighty-three were female and 72 were male. The mean weight was 164.3 g and ranged from 25 to 525 g. Five rats were neither sexed nor weighed and three rats were weighed but not sexed, and the data was classified as missing; none of these rats were RT-PCR-positive for influenza. An exact binomial test showed that neither males nor females (*p* = 0.42) were trapped at a frequency greater than would be expected by chance alone.

Eighteen of 163 rats, 11.04% (±4.81% 95CI), had swabs or lung samples that were RT-PCR positive for IAV. Nine of 161 had positive oronasal swabs (5.59 ± 3.55% 95CI), and 9 of 161 had positive paw swabs (5.59 ± 3.55% 95CI). Two rats were positive in both oronasal and paw swabs (Table 1). Therefore, the recovery of viral RNA from oronasal and paw swabs was equivalent. Only two of 108, 1.85% (±2.54% 95CI), rat lung homogenates were RT-PCR-positive for IAV. Neither individual had positive oronasal or paw swabs (Table 1).

The mean Ct value for positive samples was 36.55 (range: 34.36–42.69, STD = 1.78). The mean oronasal swab Ct value was 36.22 (STD = 0.61). The mean paw swab Ct value was 37.19 (STD = 2.43), and the mean lung Ct value was 35.14 (STD = 1.11). Mean Ct values were not significantly different between the three sample types (*p* = 0.27). All Ct values were higher than positive controls, indicating low concentrations of virus in the samples or degradation of the original sample (Table 1).

Despite our efforts, we were only able to successfully culture one sample recovered from AAF (Table 1). This sample had a Ct value of 41.61 (raw Ct value of 35.21), but sequencing was unsuccessful. None of the raw samples with positive results from initial screening could be successfully sequenced, which precluded identification of the strain or subtype.

Analysis of demographic, morphometric, and spatiotemporal data of rats using GLMs indicated that a model based on month and geocluster provided the best fit for explaining the IAV-PCR status of rats (Table 2). Month (*df* = 5, *p* = 0.002) and geocluster (*df* = 4, *p* = 0.005) accounted for 0.782 and 0.173 of the main effect, respectively. The second top-ranked model included month, geocluster, and weight; however, examination of the contribution of weight to the model indicated only a weak effect (main effect = 0.001). Therefore, we determined that the top ranked model was the most parsimonious fit for the data. Weight (*df* = 1, *p* = 1.000) and sex (*df* = 1, *p* = 1.000) of rats were also assessed as model effects but contribute only weakly to explaining the variation in the IAV-PCR status of rats.

**FIGURE 2 |:**
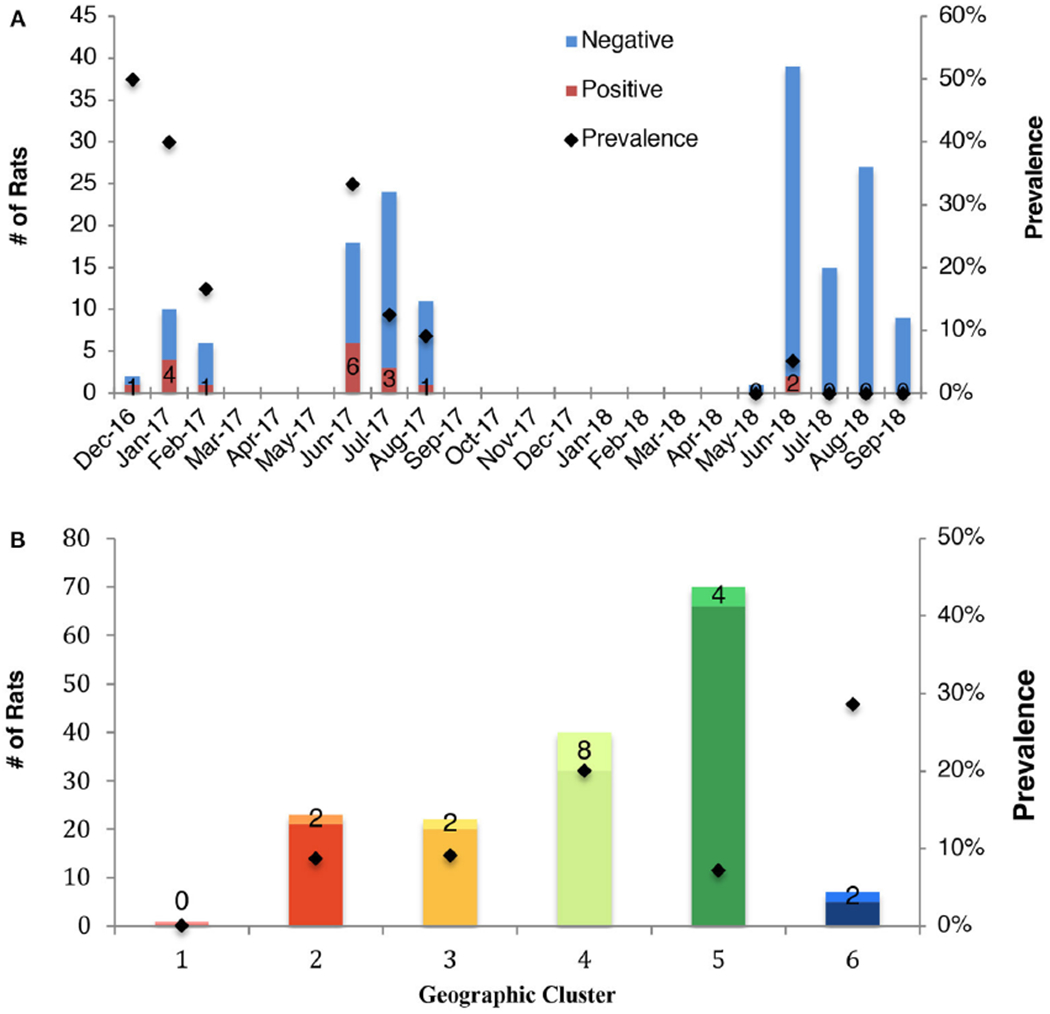
Prevalence of influenza A virus in rats according to **(A)** month of sampling (all months, including those eliminated from statistical analysis are shown), and **(B)** geocluster within the City of Boston. The total sampling effort is shown by the vertical bars. Prevalence is indicated by black marker and the number of positive rats is reported.

The effect of month on IAV status of rats indicated a seasonal signature of IAV circulation. The prevalence of IAV in rats was highest during December and January, followed by a decline over the course of the year, reaching its lowest prevalence in September (Figure 2A). The temporal changes in prevalence over the course of the study is presented (Table S1) as well as the associated odds ratios (Table S2).

A large variation in prevalence was observed between geoclusters within the City of Boston, suggesting that incidence of IAV is spatially patchy (Figure 2B). Boston Public Garden and the surrounding area (geocluster 5) were underrepresented for IAV RT-PCR positive rats (Figures 1A, 2B), whereas the neighborhoods of Brighton (geocluster 6) and Chinatown/South Boston (geocluster 4) had a higher incidence of IAV RT-PCR positive rats (Figure 2B). Therefore, spatiotemporal factors appear to be more important in determining IAV prevalence, relative to sex and weight of rats.

## DISCUSSION

The finding of IAV nucleic acid in urban Norway rats is of public health significance given the close physical proximity between humans and rats in urban environments. Rats are found in alleys, parks, subway systems, and even homes. Our study is among the first to provide evidence that rodents may play a role in the ecology of IAV in dense, urban environments.

The majority of previous studies of IAV in rodents have considered rats in rural areas following outbreaks in poultry or gamebirds and reported zero prevalence (Nettles et al., 1985; Shriner et al., 2012; Grear et al., 2016). However, lower densities of rats in rural environments may mean rats are less likely to have contact with other species, reducing the chances of infection. In the urban environment, rat populations occur at high concentrations, which may allow IAV to infect and spread within the population. Studies that have looked at rodent zoonotic pathogens in the urban environment have not reported IAV, and unbiased metagenomics studies of urban rats may have missed IAV due to the type of biospecimen analyzed, e.g., fecal pellets vs. oronasal swabs (Firth et al., 2014). We were unable to directly compare prevalence from fecal pellets and oronasal swabs, but detection of IAV from oronasal swabs in our study suggests this is an important site of the body to determine the presence of influenza in rats.

An influenza prevalence of 11.04% in the rats was unexpectedly high, given that wild rats had not been found to be PCR-positive for IAV before. This may or may not reflect the prevalence of IAV RT-PCR-positive rats across the entire city of Boston. Geographic clustering analyses revealed the Boston Public Garden and surrounding areas to be significantly underrepresented in terms of IAV RT-PCR-positive rats. While it is unknown why there were fewer IAV-positive rats in Boston Public Garden, the site is a public destination with widely dispersed waste receptacles and frequent garbage collection. Moreover, this site is not home to a permanent human population, which may limit rodent access to human waste and refuse, an important resource for rats in residential neighborhoods.

Rats are able to compensate for decreased resource density by expanding their home range (Harper and Rutherford, 2016). This phenomenon is seen in rural rats as well as those living closer to farm buildings that have significantly smaller home ranges than those living on the margins of fields (Lambert et al., 2008). Thus, it seems plausible that fewer rat interactions occur as a function of decreasing resource density, and thus fewer opportunities for transmission or mechanical vectoring of IAV may occur in public spaces relative to the residential areas of Boston. This finding is consistent with studies of rural rats that found no evidence of IAV infection and suggests that IAV prevalence in rats may be a density-dependent phenomenon (Nettles et al., 1985; Grear et al., 2016).

Sampling month was also found to be significantly associated with IAV in rats, with prevalence being highest in the winter months. Experimental studies indicate that influenza persists outside the host for longer periods at lower relative humidity and low temperatures relative to high temperatures (Weber and Stilianakis, 2008). The environmental degradation of the virus particle may be an important limiting factor in the circulation of airborne transmission typical of mammalian hosts (Pica and Bouvier, 2012). Owing to an uneven sampling effort in our study resulting in a low sample size for some months, the power of this association is unclear and may only be resolved with an enhanced study design that aims to sample rats consistently across all 12 months, rather than rely on convenience sampling.

The peak in influenza prevalence in winter observed in rats mimics the same seasonality of IAV in humans in temperate regions (Lofgren et al., 2007). However, without strain or subtype information, we cannot determine whether the seasonal pattern in rats is a reflection of endemic circulation, transmission from human sources (reverse zoonosis), or originates from other wild or domestic animals that occur in urban settings (birds, raccoons, pets, etc.). In support of the potential for reverse zoonosis, there is a growing body of literature that documents the transmission of human-origin pathogens in urban rats (Firth et al., 2014; Himsworth et al., 2015) and other peridomestic wildlife species such as skunks (Britton et al., 2010). In view of the increasing abundance of urban rodents, the incidence of zoonotic and reverse zoonotic transmission of IAV, as well as other pathogens, may become an important public health issue confronting cities.

Unfortunately, sequence data was not able to be obtained from IAV RT-PCR-positive rats in this study, likely due to very low viral titers in the original samples and degradation of viral genomes with repeated sample handling. The culturing of IAV from wild, non-avian hosts in embryonated chicken eggs (ECE) is a known challenge in the influenza field. Recent seasonal H3N2 viruses from humans have proven difficult to propagate in ECEs (Donis et al., 2014; Perez-Rubio and Eiros Bouza, 2018) and attempts to grow IAVs from marine mammals in ECEs are often unsuccessful (Puryear et al., 2016; Davis et al., in preparation). Propagation in chicken eggs depends upon the receptor binding affinity, fusion, and budding of the virus in ECEs, the concentration of virus, and the combination of both. While further passages in ECEs can result in viral isolation, using mammalian epithelial cell culture lines, such as MDCK cells, VERO cells, or in this case rat-derived epithelial cells may be beneficial in isolating and amplifying sufficient amounts of virus to be adequately sequenced (Donis et al., 2014; Perez-Rubio and Eiros Bouza, 2018). While it is unclear if rats are infected with IAV based exclusively on the molecular data presented here, the presence of viral nucleic acid in samples collected from the study population across field seasons and multiple swab sites is suggestive of replication within rats and transmission between conspecifics. Detection of viral RNA alone, however, is not conclusive evidence that the rats are truly infected. Rats may still be simply acting as mechanical vectors. This is supported by the fact that most IAV RT-PCR rats had either positive paw or oronasal swabs, but not both. Immunohistochemistry or immunofluorescence assays of infected tissue would support the role of rats acting as a host for the replication of influenza virus.

Isolation, culture, and sequencing of virus, or an unbiased metagenomics approach using oronasal or tracheal swabs, would be instrumental to learning more about the origin of these IAVs. It remains to be seen whether these viruses are rodent in origin and are endemic to rats, or if they are human in origin, picked up by rats living among urban waste. Lastly, the possibility exists that these viruses may be avian in origin given that the urban habitats where positive rats were detected are frequently shared with gulls, ducks, and pigeons. While viral sequences would be the best way to resolve this question, knowledge of rodent respiratory physiology may give us clues as to what IAV strains are most likely to affect rats. While the airway of rats has not been well characterized, the airway of mice has been (Ibricevic et al., 2006). In both human and mouse airways, α2,3-linked sialic acid receptors are found on ciliated cells and type 2 alveolar epithelial cells. These α2,3-linked sialic acid receptors preferentially bind avian IAV strains over human origin IAVs. However, unlike in humans where the α2,6-linked sialic acid receptor is expressed on both ciliated cells and goblet cells, mice have not been shown to express significant α2,6-linked sialic acid in their respiratory tract, which explains some of the difficulty in infecting mice with some human influenza strains. Assuming similar respiratory epithelial glycosylation in rats and mice, these findings suggest that the influenza strains infecting rats may not be the same as those affecting humans. Conversely, the fine detail of sialic acid linkages in the respiratory tract has proven increasingly complex, particularly with glycan array technology, and detailed mapping on rat epithelia is needed to make strong inferences about the ability of human influenza viruses to establish infection in rats in a wild setting.

The results of this study show that rats have been understudied as a potential reservoir for IAV, and that more work in this area is essential to understand the public health risks of rats and humans living at high density. To fully understand the role of rats in posing a health risk to humans or animals in an increasingly urbanized landscape, future studies should be directed at both isolating and sequencing the virus as well as larger-scale surveillance of rat populations in different urban centers.

## Supplementary Material

Table 1. Supplementary **Table S1** | The distribution of predictor/dependent variables tested with Generalized Linear Models: infection status, geocluster, sex and weight, throughout the course of the study (2016–2018).

Table 2. Supplementary **Table S2** | The odds ratios associated with prevalence for each predictor variable tested with Generalized Linear Models: month, geocluster, sex and weight.

## Figures and Tables

**TABLE 1 |:** Summary of real time-PCR-positive samples from rats.

Rat	Sex	Weight (g)	Specimen type	Original CT value	Passaged in ECEs	Post-passage PCR status	Post-passage CT value
42	Female	400	Oronasal	36.03	Yes	Negative	>45
44*	Female	300	Oronasal	35.24	Yes	Negative	>45
			Paw	36.23	Yes	Negative	>5
46	Male	400	Lung	35.93	Yes	Negative	>45
52	Male	225	Paw	37.85	No	–	–
53	Female	200	Oronasal	35.56	Yes	Negative	>45
59	Male	75	Oronasal	35.92	Yes	Negative	>45
65*	Male	150	Oronasal	36.94	Yes	Negative	>45
			Paw	35.89	Yes	Negative	>45
66	Male	90	Paw	36.90	yes	Negative	>45
71	Female	75	Paw	36.28	yes	Negative	>>45
72	Female	110	Oronasal	37.01	Yes	Negative	>45
75	Female	125	Paw	35.21	Yes	Positive	41.61
77	Male	125	Paw	42.69	No	–	–
87	Female	50	Paw	39.09	Yes	Negative	>45
94	Female	525	Oronasal	35.90	Yes	Negative	>45
98	Female	275	Paw	35.51	Yes	Negative	>45
104	Male	175	Oronasal	36.94	Yes	Negative	>45
218	Female	190	Oronasal	35.41	Yes	Negative	>45
220	Male	60	Lung	34.36	Yes	Negative	>45

Both the original cycle threshold (CT) values as well as CT values following passage in embryonated chicken eggs are reported.

* indicates the individual was positive for both oronasal and paw swabs.

**TABLE 2 |:** Best-fitting models explaining influenza A virus status of urban rats.

Model	Description			Likelihood ratio			AIC	
		*k*	*n*	log *L*	*χ* ^2^	*p*-value	Score	ΔAIC
1	Month+Geocluster*	3	160	−40.928	26.507	0.002	103.332	0.000
2	Month+Geocluster+Sex	4	154	−40.538	25.911	0.004	104.935	1.603
3	Month+Geocluster+Weight+Sex	5	154	−40.533	25.922	0.007	107.278	3.946
4	Month	2	160	−48.408	11.546	0.042	109.366	6.034
5	Geocluster	2	162	−53.152	6.727	0.151	116.688	13.356

*Number of parameters (k) and number of observations (n) included in each model are reported. The statistical significance of each model is assessed with likelihood ratio tests summarized by the log likelihood (L), Chi squared (χ^2^) and p-value. Models are ranked using Akaike’s Information Criterion (AIC) summarized by the AIC score and AIC difference (*Δ*AIC). The top model “Month + Geocluster” (indicated by an asterisk) had the lowest AIC score and p-value*.

## Data Availability

All datasets generated for this study are included in the manuscript and/or the supplementary files.

## Footnotes

1 Detailed necropsy and tissue collection procedure. http://www.vancouverratproject.com/vancouver_rat_project/results Accessed January 21,2017.
